# Altered Pharmacokinetics and Delayed Sputum Conversion in Tuberculosis Patients Co‐Infected With HIV

**DOI:** 10.1111/tmi.70165

**Published:** 2026-05-19

**Authors:** Aung Pyae Phyo, Richard M. Hoglund, Makoto Saito, Sein Sein Thi, Lei Lei Swe, Palang Chotsiri, Htet Ko Ko Aung, Win Pa Pa Tun, Banyar Maung Maung, Chanapat Pateekham, Wanitda Watthanaworawit, Gornpan Gornsawun, Nicholas J. White, Francois Nosten, Joel Tarning

**Affiliations:** ^1^ Mahidol‐Oxford Tropical Medicine Research Unit, Faculty of Tropical Medicine, Shoklo Malaria Research Unit Mahidol University Mae Ramat Tak Thailand; ^2^ Centre for Tropical Medicine and Global Health, Nuffield Department of Clinical Medicine University of Oxford Oxford UK; ^3^ Mahidol Oxford Tropical Medicine Research Unit, Faculty of Tropical Medicine Mahidol University Bangkok Thailand; ^4^ Division of Infectious Diseases University of Tokyo Tokyo Japan

**Keywords:** ethambutol, HIV‐infection, isoniazid, non‐compartmental analysis (NCA), pharmacokinetics, pulmonary tuberculosis, pyrazinamide, rifampicin, sputum conversion

## Abstract

**Background:**

HIV co‐infection affects host responses and pharmacokinetics (PK) of tuberculosis (TB) treatment. This study assessed the PK of first‐line anti‐TB drugs and clinical outcomes in patients with and without HIV co‐infection.

**Methods:**

In this prospective observational study, 61 adults with sputum‐positive pulmonary TB, either confirmed by microscopy or GeneXpert, were enrolled, including 24 with HIV co‐infection and 37 without. All HIV‐positive participants were antiretroviral‐naïve at enrolment. Standard anti‐TB therapy was given, and PK assessments were conducted on Day 1 and Week 6. Efavirenz‐based antiretroviral therapy (ART) began at a median of Day 21. Clinical outcomes, sputum conversion, and adverse events were monitored.

**Results:**

At baseline, patients with HIV co‐infection had reduced clearance of isoniazid and ethambutol, reflected by slower elimination rates. These differences resolved by Week 6 and were not significantly impacted by ART initiation. Logistic regression showed that HIV‐positive status (OR = 14.25, 95% CI: 1.22–166.37, *p* = 0.03), and higher baseline sputum bacillary load (OR = 3.92, 95% CI: 1.5–10.5, *p* = 0.006) were independently associated with delayed sputum conversion beyond 8 weeks. Baseline CRP showed an inverse association after adjustment, but this did not reach statistical significance (OR = 0.98, 95% CI: 0.96–1.00, *p* = 0.06).

**Conclusion:**

HIV co‐infection was associated with altered early PK of isoniazid and ethambutol although these differences were less apparent by Week 6. HIV‐positive status and higher baseline sputum bacillary load were associated with delayed sputum conversion. Although some pharmacokinetic differences were statistically significant, their clinical relevance remains uncertain, and the current data do not support dose adjustment. These findings suggest early pharmacokinetic variability in TB–HIV co‐infected patients and support further investigation in adequately powered exposure–response studies.

**Trial Registration:** ClinicalTrials.gov: NCT02457208

**Abbreviations**
AE: adverse event
AFB: acid‐fast bacilli
ART: antiretroviral therapy
ARV: antiretroviral
AUC_0–24_
: Area under the plasma concentration–time curve from 0 to 24 h
BMI: body mass index
CI: confidence interval
CL/F: apparent oral clearance
*C*_MAX_
: maximum plasma concentration
CONSORT: Consolidated Standards of Reporting Trials
CRP: C‐reactive protein
DAG: Directed Acyclic Graph
ETB: ethambutol
FTMEC: Faculty of Tropical Medicine Ethics Committee
GPO: Government Pharmaceutical Organization
HIV: Human Immunodeficiency Virus
HPF: high power field
INH: isoniazid
IQR: interquartile range
LLOQ: lower limit of quantification
MAC:
*Mycobacterium Avium* complex
MDR‐TB: multidrug‐resistant tuberculosis
NCA: non‐compartmental analysis
OR: odds ratio
OxTREC: Oxford Tropical Research Ethics Committee
PK: pharmacokinetics
PZA: pyrazinamide
RIF: rifampicin
SAE: serious adverse event
*t*₁/_2_
: terminal elimination half‐life
TB: tuberculosis
*T*_MAX_
: time to maximum concentration
*V/F*
: apparent volume of distribution
VIF: variance inflation factor
WHO: World Health Organization

## 1 Introduction

Tuberculosis (TB) is the leading infectious disease killer in the world, causing an estimated 1.25 million deaths in 2023. The incidence of TB remains high, with an estimated 10.8 million cases in 2023, which increased slightly from 10.7 million in 2022 and 10.1 million in 2020 [1]. Currently available standardized treatment for drug‐sensitive TB is 6 months in duration. A number of factors such as HIV status, comorbidities, malnutrition, age, sex, blood chemistry, genetics may cause variation in drug exposure and hence the treatment outcome. However, the findings are not consistent. Predictors of poor clinical outcome include older age, low body weight [2, 3, 4], anaemia, treatment delay, HIV co‐infection, non‐HIV comorbidities, extra‐pulmonary involvement [4], baseline sputum bacillary density and C‐Reactive Protein (CRP) levels [5], pharmacological exposure measures such as lower drug exposure [6, 7, 8, 9] and low CD4 counts (< 200/μL) [6]. Despite adequate dosing ensured by directly observed daily medication administration support, patients may still have suboptimal drug concentrations which may result in treatment failure or development of drug resistance [9]. Some studies did not find an association between HIV status [10], lower drug exposure and poor clinical outcome [10, 11, 12, 13].

There is also uncertainty over the risk factors associated with lower anti‐TB drug concentrations. Many studies have confirmed TB‐HIV co‐infection [14, 15, 16, 17, 18] as a risk factor for lower drug exposures but others have not [19, 20, 21, 22]. A systematic review of 27 pharmacokinetic studies reported inconsistent effects of HIV on TB drug pharmacokinetics, with some studies showing reduced drug exposure due to malabsorption or interactions among TB and HIV drugs, while others found minimal or variable effects [23]. Another systematic review of 55 studies concluded that there was a risk of suboptimal drug exposure not only in HIV‐positive individuals but also in subpopulations such as children under 2 years of age, severe malnutrition [24], and patients with specific genetic predispositions [25]. Additional factors reported to influence anti‐TB drug concentrations include sex, weight‐adjusted dosing, history of prior TB treatment, serum albumin [26], serum bilirubin [27], and the formulation and preparation of anti‐TB medications [28, 29, 30]. It is important to investigate the predictors of anti‐TB drug concentrations, particularly in HIV co‐infected patients, and to assess how these pharmacokinetic differences influence bacteriological clearance rates and overall treatment outcomes.

## 2 Materials and Methods

### 2.1 Study Design

This prospective study, was conducted in two TB treatment centres on the Thailand‐Myanmar border, compared the pharmacokinetics and pharmacodynamics of the first‐line anti‐TB drugs in adults with newly diagnosed TB with and without HIV. A total sample size of 62 (*n* = 31 in each group) was estimated to have > 80% power to detect an absolute difference of 30% in sputum conversion rates between HIV‐negative and HIV‐positive patients (Type 1 error of 5%).

### 2.2 Drug Dosing

Rimstar a four‐drug fixed‐dose combination formulation was purchased from the Government Pharmaceutical Organization (GPO) of Thailand and used consistently throughout the intensive treatment phase of the study to avoid potential variability in drug formulations. One tablet contained isoniazid (INH) (75 mg), rifampicin (RiF) (150 mg), ethambutol (ETB) (275 mg), and pyrazinamide (PZA) (400 mg). A two‐drug fixed‐dose combination formulation, containing INH (75 mg) and RiF (150 mg), from the same source was used during the continuation phase. A weight‐based once‐daily dosing scheme, as per the Thailand national guidelines and WHO guidelines, was prescribed by the treating physician. Designated health care workers were responsible for ensuring that the patients received all their doses as prescribed by observing all medication administrations. All HIV‐positive participants were antiretroviral‐naïve at enrolment. Efavirenz‐based antiretroviral (ARV) treatment was initiated at a median of 21 days (IQR: 15–27) after starting anti‐TB treatment, following national guidelines for HIV‐TB co‐infected patients in Thailand. Efavirenz was used in combination with lamivudine and either tenofovir, zidovudine, stavudine, or abacavir. No patients received protease inhibitors or pharmacokinetic booster drugs, such as ritonavir or cobicistat. Pyridoxine was co‐administered with INH according to routine clinical practice.

### 2.3 Microbiological Tests

Two sputum samples (spot and early morning) were collected for bacteriological confirmation using both conventional smear microscopy and molecular testing with GeneXpert MTB to exclude RiF‐resistant TB. All samples were stained with Ziehl‐Neelsen acid‐fast bacilli (AFB) staining for microscopic examination. The degree of AFB positivity was categorized semi‐quantitatively as follows:

Negative: No AFB in 100 fields using the 100× objective lens.

Scanty: 1–9 AFB in fields.

1+ AFB: 10–99 AFB in 100 fields.

2+ AFB: 1–10 AFB per field, check 50 fields.

3+ AFB: More than 10 AFB per field, check 20 fields.

Sputum samples positive by either smear microscopy or GeneXpert MTB were cultured for *Mycobacterium tuberculosis* on both solid and liquid media. Isolates from these cultures underwent baseline drug susceptibility testing for all first‐line anti‐TB drugs: INH, Rif, PZA, ETB, and streptomycin.

To monitor bacteriological clearance, weekly sputum smears were evaluated by microscopy and culture until conversion. Sputum culture conversion was defined as the first of three consecutive negative weekly cultures. After conversion, follow‐up sputum testing was conducted monthly until the end of the treatment course.

### 2.4 Pharmacokinetics Analysis

Blood samples (1.5 mL) for drug levels were collected at 0 (pre‐dose), 0.5, 1, 1.5, 2, 3, 4, 6, 8, 10, 12, 16, and 24 h after drug administration at drug initiation (Day 1) and then again at steady state (Week 6). Additional blood samples were collected at 6 h after dosing at months 2, 3, 4, 5, and 6. RiF, INH, PZA, and ETB plasma concentrations were quantified using high‐performance liquid chromatography linked with tandem mass spectrometry at the Clinical Pharmacology Laboratory, MORU, Bangkok. The lower limits of quantification (LLOQ) were 8.00, 12.0, 800, and 8.00 ng/mL for RiF, INH, PZA, and ETB, respectively. Quality control samples were performed in triplicate at the low, middle and high concentrations to ensure precision and accuracy during drug measurement of clinical trial samples. The total precision (i.e., relative standard deviation; % RSE) for all drug measurements was < 7.20% during drug quantification, demonstrating a robust and accurate drug quantification process.

Individual concentration‐time data were evaluated using a non‐compartmental analysis approach (NCA) in PKanalix version 2024R1 (Lixoft, Antony, France). This approach was chosen because of the modest sample size and the study's primary objective of descriptive comparison of drug exposure between groups. Total exposure up to 24 h after dose (AUC_0–24_) was calculated using the linear trapezoidal method for ascending concentrations and the logarithmic trapezoidal method for descending concentrations. The terminal elimination constant (*λ*_Z_) was estimated by log‐linear regression (with at least 3 datapoints in the elimination phase) of the observed concentration‐time data in the terminal elimination phase. The terminal elimination half‐life (t_1/2_) was calculated by (ln2)/*λ*_Z_. Peak concentration (*C*_MAX_) and time to peak concentration (*T*_MAX_) were taken directly from the observed data. Apparent oral volume of distribution (*V/F*) and oral elimination clearance (CL*/F*) were computed individually according to standard equations [Equations (1) and (2)]. *F* is the fraction of drug absorbed.

$$\mathrm{CL}/F=\frac{\text{dose}}{{\mathrm{AUC}}_{\inf}}\;\left(\text{first dose}\right);$$

(1) $$\mathrm{CL}/F=\frac{\text{dose}}{{\mathrm{AUC}}_{0-24,\mathrm{SS}}}\;\left(\text{steady}-\text{state}\right)$$

(2) $$V/F=\frac{\mathrm{CL}/F}{\lambda_{z}}\;\left(\text{first dose}\&\text{steady}-\text{state}\right)$$

where dose is the individual daily dose and AUC_*inf*_ is the AUC extrapolated to infinity from the last predicted concentration at the last time point. Datapoints that fell under the LLOQ before *T*_MAX_ was imputed as zero and observed data after *T*_MAX_ that fell under LLOQ was coded as missing for estimation. One patient had the last sample collected at 6 h after dose and could therefore not fully characterize the terminal elimination phase and was therefore excluded from the analysis. Statistical comparisons were carried out with a Wilcoxon signed rank test. For comparison between HIV‐positive and HIV‐negative the test was a non‐paired test (equal to a Mann–Whitney *U*‐test). For comparisons between initiation and steady‐state a paired test was used and patients with data from just one phase were excluded.

### 2.5 Statistical Analysis

For comparing continuous variables between groups, *t*‐tests were used for normally distributed data. For categorical variables, Chi‐square or Fisher's exact tests were applied as appropriate. Adverse events (AEs) were analysed using rates calculated as the number of episodes divided by person‐time (in days), expressed as episodes per 100 person‐days. Rates were compared between HIV‐positive and negative groups using Poisson regression.

Delayed sputum conversion by Week‐8 was analysed using logistic regression, with HIV status as the primary exposure. The multivariable model was adjusted for covariates that were identified based on a directed acyclic graph (DAG) including baseline sputum bacillary load and CRP levels. Multicollinearity was assessed using variance inflation factors. All statistical analyses were conducted in Stata version 16.

## 3 Results

### 3.1 Baseline Characteristics

**FIGURE 1:**
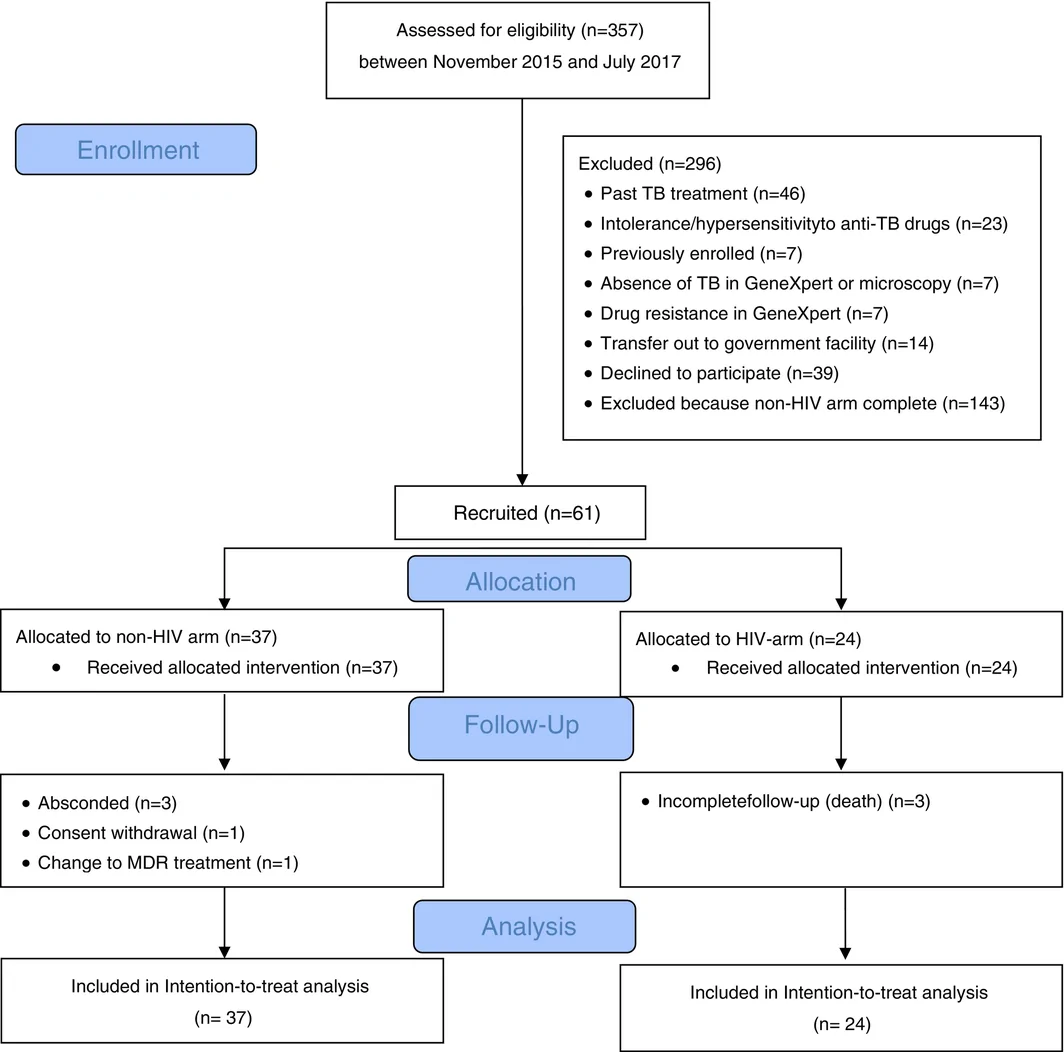
CONSORT flow diagram of participant recruitment and follow‐up.

This study was conducted between November 2015 and July 2017. Among 357 newly diagnosed TB patients assessed for eligibility, 61 patients were willing to participate and were enrolled in this study: 37 patients without HIV and 24 patients with HIV. The initial plan was to recruit 62 patients: 31 HIV negative and 31 HIV positive with smear confirmed pulmonary TB. However, the recruitment process was slower than expected, with the HIV negative arm taking 4 months to complete while only 24 HIV positive patients could be enrolled over a period of 20 months. To compensate for this, additional patients were recruited in the HIV negative arm. The main reasons for exclusion were refusal to participate (*n* = 39), received TB treatment in the past (*n* = 46), previous intolerance/hypersensitivity to anti‐TB drugs (*n* = 23) and HIV‐negative status as the non‐HIV arm completed earlier (*n* = 143) (Figure 1, CONSORT chart). The majority of patients were of Burmese (52.5%, 32/61) and Karen (29.5%, 18/61) ethnic origins. Patients co‐infected with HIV were younger than those without (mean age of 37.9 vs. 43.6 years) and 66.7% (16/24) were Burmese. Compared with HIV‐negative participants, HIV‐positive participants had lower BMI, less cavitary disease on chest radiography, lower baseline bacillary density by microscopy, and slightly lower eGFR although eGFR remained within the normal range in both groups (Table 1). There was no significant difference in weight‐normalized anti‐TB dosing (mg/kg) between patients with HIV and those without (Table 1). There were no missed doses and there was no vomiting.

**TABLE 1:** Demographic, epidemiological and clinical characteristics of recruited patients.

	Patients without HIV (*n* = 37)	Patients with HIV (*n* = 24)	*p*
Demographic (Baseline)			
Male	24/37 (64.9%)	17/24 (70.8%)	0.63
Age (year)	46.0 (32.0–52.0)	38 (31.5–41.5)	0.08
BMI (kg/m^2^)	18.5 (17.2–19.8)	17.3 (16.4–18.2)	0.04
Body temperature (°C)	37.0 (37.0–38.0)	37.0 (37.0–38.0)	0.90
Smoker (ex‐ or current)	15/37 (40.5%)	9/24 (37.5%)	0.81
Chest Radiograph (Baseline)			
Baseline CXR done	34	23	
Cavitation			**0.006**
No cavitation *n* (%)	22/34 (64.7)	22/23 (95.7)	
Unilateral cavitation *n* (%)	4/34 (11.8)	0/23 (0)	
Bilateral cavitation *n* (%)	8/34 (23.5)	1/23 (4.3)	
Opacity			**0.07**
No opacity *n* (%)	17/34 (50.0)	17/23 (73.9)	
Unilateral opacity *n* (%)	2/34 (5.9)	2/23 (8.7)	
Bilateral opacity *n* (%)	15/34 (44.1)	4/23 (17.4)	
Blood parameters (Baseline)			
Blood test done	37	24	
C‐Reactive Protein (mg/L)	57.0 (38.7–72.9)	134.0 (60.3–> 200)	**0.001**
Fasting blood glucose (mg/dL)	87 (80.0–97.0)	90.5 (85.0–97.0)	0.36
Haemoglobin (g/dL)	10.8 (9.4–11.8)	8.3 (6.5–11.4)	**0.008**
Serum Creatinine (mg/dL)	0.7 (0.6–0.7)	0.85 (0.7–1.5)	**0.0007**
eGFR	111.6 (99.2–127.2)	104.3 (55.4–121.0)	**0.02**
CD4 count	Not done	41 (21–118)	**NA**
Comorbidity			
Any comorbidities	5/37 (13.5%)	4/24 (16.7%)	
Below knee amputation	1	0	
Depression (Moderate)	0	1	
Hearing loss (Unilateral)	1	0	
Injury	1	0	
Lymphadenitis	0	2	
Oral candidiasis	0	1	
Peripheral neuropathy	1	0	
Arthritis	1	0	
Severe pneumonia	0	3	
Drug dosing (mg/kg body weight)			0.39
Isoniazid	4.9 (4.5–5.2)	5.0 (4.8–5.3)	0.39
Rifampicin	9.8 (9.0–10.5)	10.0 (9.7–10.6)	0.39
Pyrazinamide	26.1 (24.0–27.9)	26.7 (25.8–28.2)	0.39
Ethambutol	17.9 (16.5–19.2)	18.3 (17.7–19.4)	0.39
Baseline bacteriological result by microscopy			**0.0003**
*N* tested	37	24	
AFB Negative	1/37 (2.7%)	5/24 (20.8%)	
1–9 AFB/100 HPF	4/37 (10.8%)	5/24 (20.8%)	
10–99 AFB/100 HPF	1/37 (2.7%)	4/24 (16.7%)	
1–10 AFB/1 HPF	5/37 (13.5%)	3/24 (12.5%)	
> 10 AFB/1 HPF	26/37 (70.3%)	7/24 (29.2%)	
Drug sensitivity			
MDR TB^a^	1/37 (2.7%)	0/24 (0.0%)	1.00

*Note:* eGFR = estimated glomerular filtration rate (mL/min/1.73 m^2^), calculated using the CKD‐EPI equation based on serum creatinine, age, and sex. *n* (%) or median (interquartile range) is shown. *p* values are calculated by Wilcoxon rank‐sum test. Bold values indicates *p* < 0.05. BMI 0.04, Cavitation 0.006, CRP 0.001, Haemoglobin 0.008, Serum Creatinine 0.0007, eGRF 0.02, Baseline bacteriological result by microscopy 0.0003.

Abbreviations: AFB = Acid Fast Bacilli, BMI = body mass index, HPF = High Power Field, MDR = multi‐drug resistance.

^a^ Sputum culture result came back on week‐3 as MDR TB.

### 3.2 Pharmacokinetic Parameters by HIV Status

On Day 1, the pharmacokinetic properties of all four first‐line anti‐TB drugs were overall similar between HIV‐positive and HIV‐negative patients, except for the terminal elimination half‐lives, which was significantly longer in the HIV‐positive group (*p* < 0.05, Table 2). In addition, the absorption of RiF and ETB was significantly slower in the HIV‐positive group (*p* < 0.05). Overall drug exposures were similar between HIV‐positive and HIV‐negative patients (i.e., < 15% relative difference) except for INH which had a 39% higher drug exposure in HIV‐positive patients.

**TABLE 2:** Pharmacokinetic parameter of rifampicin, isoniazid, pyrazinamide and ethambutol in HIV‐positive and HIV‐negative patient during the initiation phase (Day 1) and at steady state (Week 6).

Pharmacokinetic parameters	Initiation phase (Day 1)			Steady state (Week 6)		
	HIV‐positive (*n* = 24)	HIV‐negative (*n* = 36)	*p*	HIV‐positive (*n* = 21)	HIV‐negative (*n* = 35)	*p*
Rifampicin						
*C*_MAX_ (ng/mL)	7330 (5530–8610)	7620 (6160–9090)	0.17	6950 (4920–8410)	7840 (5720–8980)	0.13
*T*_MAX_ (h)	3.00 (2.00–3.31)	2.00 (1.50–2.02)	**0.035**	2.15 (2.00–3.00)	2.00 (1.50–3.00)	0.076
AUC_0–24_ (h × μg/mL)	66.6 (56.5–79.4)	59.0 (44.9–73.2)	0.36	33.6 (28.5–41.6)	34.0 (25.3–47.7)	0.65
*t*_1/2_ (h)	5.40 (3.98–7.2)	3.37 (2.58–4.18)	**< 0.0001**	2.29 (2.14–3.49)	2.08 (1.60–2.37)	**0.0067**
CL/*F* (L/h)	6.42 (4.76–8.15)	7.35 (5.97–10.2)	0.18	13.2 (10.6–15.8)	13.5 (9.44–17.9)	0.96
*V/F* (L)	48.6 (38.1–57.5)	36.4 (30.1–51.7)	**0.012**	50.3 (40.3–67.1)	39.6 (33.0–50.0)	**0.0048**
Isoniazid						
*C*_MAX_ (ng/mL)	4930 (4040–5580)	4850 (3500–6090)	1.00	4340 (3610–5050)	4050 (3180–5070)	0.83
*T*_MAX_ (h)	1.00 (0.515–1.50)	1.00 (0.500–1.50)	0.30	1.50 (0.500–2.37)	1.00 (0.510–1.50)	0.41
AUC_0–24_ (h × μg/mL)	21.5 (15.0–29.3)	15.5 (9.69–21.3)	**0.017**	14.9 (11.7–21.8)	13.5 (9.65–18.3)	0.45
*t*_1/2_ (h)	4.41 (4.18–5.60)	3.92 (3.56–4.34)	**0.00024**	3.79 (3.55–4.07)	3.79 (3.33–4.14)	0.80
CL/*F* (L/h)	10.5 (7.28–14.4)	15.0 (10.5–20.4)	**0.0064**	15.1 (10.8–18.4)	15.9 (12.6–23.3)	0.31
*V/F* (L)	69.9 (52.6–97.5)	77.5 (57.2–106)	0.43	77.8 (56.7–106)	84.3 (70.4–116)	0.40
Pyrazinamide						
*C*_MAX_ (μg/mL)	33.9 (31.8–39.1)	37.6 (32.6–41.2)	0.20	37.8 (32.9–42.6)	37.1 (33.0–43.1)	0.72
*T*_MAX_ (h)	1.50 (0.880–2.27)	1.09 (0.528–2.00)	0.42	1.50 (1.00–2.38)	1.48 (1.00–2.00)	0.35
AUC_0–24_ (h × μg/mL)	376 (319–405)	352 (289–398)	0.40	332 (264–430)	310 (250–365)	0.30
*t*_1/2_ (h)	8.63 (7.47–10.9)	7.40 (6.55–8.10)	**0.01**	6.30 (5.68–7.75)	6.00 (5.38–6.91)	0.35
CL/*F* (L/h)	2.83 (2.34–3.47)	2.94 (2.49–3.90)	0.27	3.61 (2.75–4.21)	3.88 (3.39–4.93)	0.10
*V/F* (L)	35.7 (31.9–38.7)	31.5 (28.4–36.4)	**0.048**	30.3 (27.7–35.1)	32.9 (29.0–38.7)	0.28
Ethambutol						
*C*_MAX_ (ng/mL)	1520 (1370–2200)	2140 (1610–2720)	0.12	2470 (1830–3650)	2590 (1800–3630)	0.92
*T*_MAX_ (h)	3.08 (2.00–4.00)	2.1 (2.00–3.00)	**0.0073**	3.00 (2.15–4.00)	3.00 (2.00–3.03)	**0.043**
AUC_0–24_ (h × μg/mL)	12.0 (9.70–18.2)	11.4 (9.19–13.3)	0.16	16.9 (13.7–20.0)	16.7 (13.4–21.6)	0.80
*t*_1/2_ (h)	10.2 (8.78–12.3)	8.87 (7.90–10.6)	**0.022**	13.8 (9.13–15.1)	13.5 (12.2–16.1)	0.36
CL/*F* (L/h)	36.4 (27.3–62.9)	49.1 (40.5–57.5)	**0.037**	36.0 (27.6–44.4)	37.5 (31.3–45.7)	0.58
*V/F* (L)	658 (400–782)	600 (514–768)	0.98	699 (470–818)	706 (556–1010)	0.21

*Note:* Data are shown as median (interquartile range). *p* values were calculated using the non‐parametric Mann–Whitney *U*‐test. Bold values indicates *p* < 0.05. Rifampicin 0.035, < 0.0001, 0.0067, 0.012, 0.0048, Isoniazid: 0.017, 0.00024, 0.0064, Pyrazinamide: 0.01, 0.048, Ethambutol: 0.0073, 0.043, 0.022, 0.037.

At presumed steady state (Week 6) HIV‐positive patients had a longer RiF terminal elimination half‐life (*p* < 0.05) and a higher (*V/F*) than HIV‐negative patients (Table 2 and Figure S1). A significant difference in time to maximum concentration of ETB was also observed, even if the median were the same. All HIV‐positive patients had already started their ART by Week 6.

### 3.3 Changes in Pharmacokinetic Parameters Over Time

**FIGURE 2:**
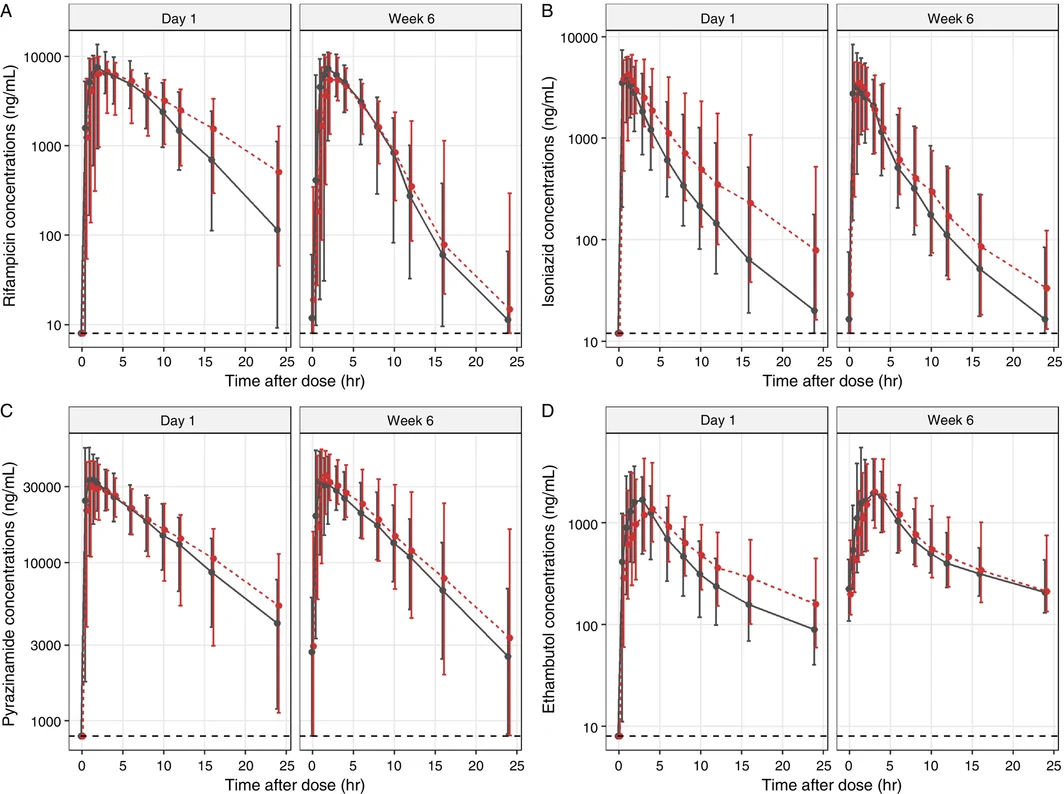
Median venous plasma concentration‐time curves of rifampicin (A), isoniazid (B), pyrazinamide (C), and ethambutol (D) in patients with HIV (dashed red lines) and those without (solid black lines). Error bars indicate the 90% confidence interval.

At steady‐state, daily drug exposure (AUC_0–24_) and half‐life were both reduced by approximately 40%, largely as a result of doubling of oral clearance (CL/*F*) (Figure 2A). These changes could reflect enzymatic induction by RiF itself as well as combined induction effects of RiF and efavirenz.

For INH and PZA, pharmacokinetic parameters also differed between the two phases. Peak concentrations (*C*_max_) of INH, daily exposures (AUC_0–24_) of INH, and terminal half‐lives (*t*_1/2_) for both drugs were reduced, while oral clearance (CL*/F*) increased at 6 weeks. The *V/F* were higher at 6 weeks for INH (Figure 2B,C and Table S1).

In contrast, ETB showed a shorter terminal elimination half‐life during the initiation phase compared to steady state, primarily because of increased oral clearance at the start of treatment. This resulted in a higher exposure (both AUC and *C*max) at 6‐week (Figure 2D).

### 3.4 Factors Affecting Total Drug Exposure

The regression analysis (Tables S2 and S3) at Day 1 and week‐6 focused on identifying factors influencing AUC_0–24_, as significant variability between HIV‐positive and HIV‐negative patients was observed during treatment initiation. AUC_0–24_ was selected as the primary pharmacokinetic exposure metric because it provides a comprehensive measure of cumulative drug exposure over a defined time, and therefore most likely reflects the pharmacokinetic driver of therapeutic success. This parameter is more clinically relevant for assessing drug efficacy and safety compared to other pharmacokinetic metrics, as it reflects total drug exposure and is derived directly from observed concentration‐time data.

For INH, HIV‐positive status was a significant predictor of higher AUC_0–24_ in both univariable and multivariable analyses and resulted in a substantial difference in drug exposure. Also, ETB demonstrated a significant association between HIV‐positive status in the multivariable model, but the magnitude of this difference was small (< 10%) and of no clinical importance. For ETB, HIV‐positive status showed a marginal association at baseline (*p* = 0.05), but this did not persist at Week 6. Male sex and BMI were significant predictors for RiF and INH, with males showing lower AUC_0‐24_ than females for both RiF and INH. These sex‐related differences remained statistically significant at both timepoints for RiF. At week‐6, these sex‐related differences remained significant, particularly for RiF, where males had consistently lower AUC_0–24_ values. BMI showed a dual impact, being associated with lower AUC_0–24_ for RiF and higher AUC_0‐24_ for INH.

CRP also emerged as an independent predictor of RiF AUC_0–24_ at Week 6, with higher CRP associated with significantly increased exposure (β = 0.50, *p* = 0.04), possibly reflecting inflammation‐induced changes in hepatic drug metabolism.

These findings suggest that the HIV status, systemic inflammation, sex, and nutritional status could contribute to variability in anti‐TB drug exposure, particularly during treatment initiation.

### 3.5 Clinical Outcome

#### 3.5.1 Bacteriological Parameters

Baseline sputum bacilli densities before initiating treatment were 33/61 patients (54.1%) with 3+, 8/61 (13.1%) with 2+, 5/61 (8.2%) with 1+, and 9/61 (14.8%) with less than 1+ (scanty). AFB were not detected by microscopy in six patients (9.8%, 6/61) but were identified by GeneXpert testing. HIV‐positive patients tended to have lower bacillary densities; 10/24 (41.7%) were either negative or scanty compared to 5/37 (13.5%) in non‐HIV patients (*p* = 0.01), which is consistent with prior studies [31, 32]. This finding also corresponded to fewer HIV‐positive patients exhibiting pulmonary pathology, such as cavitation or opacities on chest X‐rays.

Sputum conversion data were available for 56/61 patients (91.8%), as study participation was terminated before sputum conversion occurred in five patients (withdrawal of consent (*n* = 1), change in TB treatment due to MDR‐TB infection (*n* = 1), and death (*n* = 3)). The median time for sputum conversion by culture after starting treatment was 6.5 weeks. At Week 4, 21/56 patients (37.5%, 95% CI: 24.9–51.5) became culture negative, and by Week 8, 40/56 patients (71.4%, 95% CI: 57.8–82.7) had achieved culture negativity.

To assess the association between co‐variates and bacteriological delayed bacteriological clearance at Week 8, multivariable logistic regression was performed adjusting for confounders including baseline bacillary density, and pharmacokinetic exposure parameters (Table 3).

**TABLE 3:** Logistic regression analysis for delayed sputum clearance (by Culture) at Week 8.

Variable^a^	Univariable	*p*	Multivariable^b^	p
	OR (95% CI)		OR (95% CI)	
HIV‐positive	1.30 (0.40–4.25)	0.67	14.25 (1.22–166.37)	**0.03**
Age (per 10 years)	1.39 (0.87–2.22)	0.17	1.88 (0.89–3.95)	0.09
BMI	0.84 (0.63–1.12)	0.23	0.79 (0.52–1.20)	0.26
Male	2.00 (0.54–7.40)	0.30	0.82 (0.13–5.03)	0.83
log_Rif AUC_0–24_ D1	0.59 (0.03–10.47)	0.72	0.35 (0.04–2.81)	0.33
log_INH AUC_0–24_ D1	0.82 (0.06–10.66)	0.88	3.96 (0.45–34.85)	0.21
log_PZA AUC_0–24_ D1	0.13 (0.000–50.91)	0.50	0.58 (0.011–30.34)	0.79
log_ETB AUC_0–24_ D1	1.36 (0.06–30.23)	0.85	0.47 (0.05–4.63)	0.52
Smoking	1.17 (0.36–3.81)	0.80	3.02 (0.54–17.00)	0.21
CRP baseline	0.99 (0.98–1.00)	0.35	0.98 (0.96–1.00)	**0.06**
Bacillary density^c^	2.06 (1.27–3.35)	**0.003**	3.92 (1.47–10.50)	**0.006**

*Note:* Bold values indicates *p* < 0.05. HIV‐positive 0.03, Bacillary density 0.006.

^a^ At TB diagnosis (i.e., baseline).

^b^ VIF values for the multivariable model range between 1.11 and 2.06, indicating no significant multicollinearity between included variables.

^c^ An ordered categorical variable based on semi‐quantified baseline sputum bacillary density by microscopy was handled as a continuous variable.

In the logistic regression analysis, baseline sputum bacillary density emerged as the only significant predictor in the univariable model (OR 2.06, 95% CI: 1.27–3.35, *p* = 0.003) and remained significant in the multivariable model (OR 3.92, 95% CI: 1.47–10.50, *p* = 0.006), underscoring its strong association with delayed sputum clearance. HIV‐positive status was not significant in the univariable model (OR 1.30, 95% CI: 0.40–4.25, *p* = 0.67) but became strongly associated in the multivariable model (OR = 14.25, 95% CI: 1.22–166.37, *p* = 0.03), suggesting an increased risk of delayed sputum conversion due to immune suppression and treatment complexities.

The inclusion of baseline CRP as a covariate in the regression model contributed to this increase in the odds ratio for HIV‐positive status. Without CRP, the odds ratio for HIV‐positive status was 6.05 (*p* = 0.10) but rose to 14.26 after accounting for CRP. After adjustment, baseline CRP showed an inverse association with delayed sputum conversion, but this did not reach conventional statistical significance (OR 0.98, 95% CI: 0.96–1.00, *p* = 0.06). Variance inflation factor analysis showed no important multicollinearity among included covariates, with VIF values ranging from 1.11 to 2.06.

Variable selection for the regression model was guided by a DAG (Figure S2), which identified baseline bacillary density and CRP as key covariates relevant to delayed sputum conversion. CRP was retained in the model due to its biological relevance as a marker of systemic inflammation.

#### 3.5.2 AEs

The overall AE incidence rate was 1.35 episodes per 100 person‐days, with HIV‐positive patients experiencing a slightly higher rate (1.45 per 100 person‐days) compared to HIV‐negative patients (1.28 per 100 person‐days). Gastrointestinal, dermatological, and respiratory complaints were the three most common AE categories. However, none of these subgroups showed statistically significant differences in adjusted Poisson regression analysis between HIV‐positive and HIV‐negative groups. Four episodes of serious adverse events (SAEs) were reported in the HIV‐positive group, including three deaths, attributed to septicaemia from aspiration pneumonia, suspected *Mycobacterium avium* complex (MAC) infection, and multi‐organ failure. No SAEs or deaths occurred in the HIV‐negative group.

These findings suggest a greater overall burden of AEs among HIV‐positive patients, though subgroup analyses were limited by small numbers and did not identify any statistically significant differences.

## 4 Discussion

This study shows that the pharmacokinetics of first‐line anti‐TB antibiotics are influenced by multiple factors, including HIV status, particularly during the treatment initiation phase. Specifically, HIV‐positive status was associated with higher exposure to INH, and these findings are consistent with prior reports of altered drug pharmacokinetics in HIV co‐infected patients. All drugs also showed longer terminal elimination half‐life in HIV‐positive patients. The mechanisms underlying these differences are uncertain and may reflect a combination of systemic inflammation, immune suppression, differences in hepatic or renal function, or other unmeasured physiological factors rather than a single HIV‐specific pathway.

Despite these pharmacokinetic differences, the current clinical strategy of starting ARV therapy after the initiation of anti‐TB treatment remains appropriate, as the early pharmacokinetic variations appeared less pronounced by Week 6. The observed temporal changes likely reflect combined effects of RiF and efavirenz rather than RiF autoinduction alone. These findings suggest that HIV‐associated pharmacokinetic changes do not necessarily undermine the efficacy of current treatment protocols in co‐infected patients. However, because RiF reduces efavirenz levels by approximately 22%, potential drug–drug interactions remain clinically relevant and should be considered when interpreting treatment response in TB–HIV co‐infected patients.

Comparing total exposure at Day 1 and at Week 6 showed that the total exposure of RiF is significantly lower at Week 6, likely reflecting induction of drug metabolism. INH also showed a significantly lower total exposure at Week 6. This has been identified before and have been proposed to be linked to the total RiF dose. On the other hand, ETB showed a higher total exposure, this could possibly be due to differences in apparent clearance and *V/F* between sampling timepoints.

In logistic regression analysis, HIV‐positive status and baseline sputum bacillary density were strong independent predictors of delayed sputum clearance at Week 8. Baseline CRP showed an inverse association after adjustment, but this did not reach conventional statistical significance in the revised model. The adjusted odds ratio of 0.98 per 1 mg/L increase suggests a small decrease in the odds of delayed conversion with higher CRP, although this finding should be interpreted cautiously. This contrasts with prior reports in which higher CRP was associated with delayed conversion [5, 33]. In this cohort, CRP may partly reflect a different inflammatory phenotype or disease presentation, but the limited sample size and attenuation of the association after re‐analysis mean that no strong inference should be drawn. The predictive effect of HIV on delayed clearance became evident only after adjusting for baseline inflammation and bacillary burden, underscoring the need to account for confounding effects to reveal the true impact of HIV‐related immune suppression. These findings are consistent with established evidence that both immune suppression and initial mycobacterial load influence bacteriological response to TB therapy [3, 34].

Although pharmacokinetic alterations were observed in the HIV‐positive group, further studies are needed to determine whether these differences have clinically meaningful implications for bacteriological clearance or treatment optimisation.

## 5 Limitations

The study has many limitations. First, the smaller‐than‐planned sample size (24 HIV‐positive and 37 HIV‐negative), which reduced the statistical power from 80% to approximately 72% to detect the same 30% difference. The study may therefore have been underpowered to detect more modest differences in sputum conversion rates between groups. Second, genetic determinants of INH pharmacokinetics, including NAT2 polymorphisms, were not assessed. Third, the immune and inflammatory profiling beyond CRP was limited, which may restrict understanding of the full scope of inflammatory and metabolic changes in co‐infected patients. Finally, delayed initiation of ARV treatment, in line with guidelines, may have limited insights into potential drug–drug interactions during the early treatment phase.

## 6 Conclusion

This study suggests that HIV status and higher baseline sputum bacillary density are associated with delayed sputum conversion. HIV co‐infection was also associated with altered early pharmacokinetics of INH and ETB, although these differences were less apparent by Week 6. Larger studies are needed to determine whether these pharmacokinetic differences have clinically meaningful implications for treatment response.

## Funding

This study was funded by the Wellcome Trust where the funder had no role in the design, data collection, analysis, interpretation, or writing of the manuscript.

## Ethics Statement

This study was approved by Oxford Tropical Research Ethics Committee (OxTREC), the Ethics Committee of the Faculty of Tropical Medicine (FTMEC), Mahidol University.

## Consent

Written informed consent was obtained from all participants prior to enrolment.

## Conflicts of Interest

The authors declare no conflicts of interest.

## Supporting information


**Table S1:** Pharmacokinetic parameter (median and IQR) of rifampicin, isoniazid, pyrazinamide and ethambutol between the initiation phase (Day 1) and steady state (Week 6).
**Table S2:** Determinants of AUC_0–24_ of antiTB drugs at baseline by univariable and multivariable analyses‐1.
**Table S3:** Determinants of AUC_0–24_ of antiTB drugs at week‐6 by univariable and multivariable analyses.

**Figure S1:** Comparisons of PK parameters in patients with HIV and those without: (A, D, G, J) peak concentration, (B, E, H, K) daily exposure, and (C, F, I, L) terminal half‐life. Each row represents the pharmacokinetic parameters of each anti‐TB drug: (A–C) rifampicin, (D–F) isoniazid, (G–I) pyrazinamide, and (J–L) ethambutol. *p* values were calculated using the non‐parametric Mann–Whitney *U*‐test.

**Figure S2:** Directed Acyclic Graph (DAG) illustrating the hypothesized relationships between HIV status, baseline CRP, sputum load, drug level, and delayed sputum clearance.

**Figure S3:** Individual concentration‐time profiles. Thin lines are individual profiles. The thick line is the median profile for each group. The red curves are HIV negative and blue are HIV positive.

## Data Availability

The data supporting the findings of this study are available in accordance with the data sharing policy of the Mahidol Oxford Tropical Medicine Research Unit (MORU). Researchers may request access by contacting the MORU Data Access Committee datasharing@tropmedres.ac. Requests will be evaluated based on scientific merit and ethical considerations.
