# Supplementary material for: Altered Pharmacokinetics and Delayed Sputum Conversion in Tuberculosis Patients Co‐Infected With HIV
Source: Trop Med Int Health. 2026 May 19;31(9):1261–72. doi: 10.1111/tmi.70165 (PMC13533638; doi:10.1111/tmi.70165)
Supplement: Supplementary file 1 [file TMI-31-1261-s004.docx]

**Table S1** Pharmacokinetic parameter of rifampicin, isoniazid, pyrazinamide and ethambutol between the initiation phase (day1) and steady state (week 6).

| Pharmacokinetic parameters | Initiation phase (day 1)  (n = 56) | Steady state (week 6)  (n = 56) | P-value |
| --- | --- | --- | --- |
| Rifampicin |  |  |  |
| C_MAX_ (ng/mL) | 7620 (6120, 9030) | 7430 (5560, 8760) | 0.18 |
| T_MAX_ (hr) | 2.00 (1.52, 3.00) | 2.00 (1.50, 3.00) | 0.41 |
| AUC_0-24_ (hr×µg/mL) | 61.2 (47.0, 79.0) | 33.8 (25.3, 46.2) | **<0.0001** |
| t_1/2_ (hr) | 3.75 (3.14, 5.44) | 2.22 (1.70, 2.63) | **<0.0001** |
| CL/F (L/hr) | 6.66 (5.02, 9.65) | 13.3 (9.94, 17.2) | **<0.0001** |
| V/F (L) | 38.3 (31.8, 54.4) | 41.8 (34.2, 51.9) | 0.58 |
| Isoniazid |  |  |  |
| C_MAX_ (ng/mL) | 4930 (3880, 5910) | 4200 (3240, 5060) | **0.0099** |
| T_MAX_ (hr) | 1.00 (0.500, 1.05) | 1.00 (0.500, 1.66) | 0.027 |
| AUC_0-24_ (hr×µg/mL) | 16.4 (12.0, 24.7) | 13.6 (10.1, 20.9) | **<0.0001** |
| t_1/2_ (hr) | 4.02 (3.69, 4.43) | 3.79 (3.35, 4.12) | **0.0014** |
| CL/F (L/hr) | 11.9 (9.04, 18.0) | 15.2 (11.1, 22.3) | **<0.0001** |
| V/F (L) | 72.2 (53.7, 100) | 82.0 (61.3, 113) | **0.0030** |
| Pyrazinamide |  |  |  |
| C_MAX_ (µg /mL) | 34.6 (31.6, 40.8) | 37.4 (32.8, 43.0) | 0.099 |
| T_MAX_ (hr) | 1.09 (0.529, 2.00) | 1.50 (1.00, 2.00) | 0.46 |
| AUC_0-24_ (hr×µg/mL) | 352 (296, 396) | 327 (253, 397) | 0.16 |
| t_1/2_ (hr) | 7.76 (6.55, 8.64) | 6.03 (5.39, 7.03) | **<0.0001** |
| CL/F (L/hr) | 2.90 (2.47, 3.74) | 3.68 (3.18, 4.48) | **<0.0001** |
| V/F (L) | 32.4 (28.6, 37.7) | 32.3 (28.2, 37.0) | 0.31 |
| Ethambutol |  |  |  |
| C_MAX_ (ng/mL) | 1880 (1410, 2670) | 2500 (1820, 3660) | **0.0011** |
| T_MAX_ (hr) | 3.00 (2.00, 3.04) | 3.00 (2.00, 4.00) | 0.27 |
| AUC_0-24_ (hr×µg/mL) | 11.4 (9.19, 13.7) | 16.8 (13.4, 21.5) | **<0.0001** |
| t_1/2_ (hr) | 9.32 (8.03, 10.9) | 13.5 (11.9, 16.0) | **<0.0001** |
| CL/F (L/hr) | 46.2 (35.1, 62.9) | 36.4 (30.9, 45.6) | **0.00011** |
| V/F (L) | 607 (489, 777) | 703 (532, 945) | 0.137 |

Values are presented as medians (IQRs) P-values were calculated using the non-parametric paired Wilcoxon signed rank test. Patients with data from just one occasion was ignored.

| **Table S2** Determinants of AUC_0-24_ of antiTB drugs at baseline by univariable and multivariable analyses | | | | |  | |
| --- | --- | --- | --- | --- | --- | --- |
| **Variable** | **Univariable β* (95% CI)** | **p, value** | **Multivariable β* (95% CI)** | **p-value** | |  |
| **Rifampicin AUC_0-24_** |  |  |  |  | |  |
| HIV positive | 8.82 (-2.55, 20.20) | 0.13 | 10.25 (-2.59, 23.09) | 0.11 | |  |
| Age (per 10 years) | -0.27 (-4.93, 4.39) | 0.91 | 4.21 (-0.30, 9.59) | 0.09 | |  |
| BMI | -2.55 (-4.64, -0.47) | **0.02** | -3.01 (-5.19, -0.84) | **0.008** | |  |
| Male | -11.77 (-23.45, -0.087) | **0.048** | -16.18 (-28.02, -4.33) | **0.008** | |  |
| C-Reactive Protein | 0.03 (-0.05, 0.12) | 0.44 | 0.03 (-0.06, 0.13) | 0.51 | |  |
| Smoking | 5.90 (-5.75, 17.56) | 0.31 | 3.09 (-7.84, 14.03) | 0.57 | |  |
| Creatinine | 1.31 (-3.30, 5.91) | 0.57 | -2.54 (-7.23, 2.15) | 0.28 | |  |
| **Isoniazid AUC_0-24_** |  |  |  |  | |  |
| HIV positive | 8.43 (3.09, 13.78) | **0.003** | 9.72 (3.80, 15.64) | **0.002** | |  |
| Age (per 10 years) | -1.43 (-4.15, 0.81) | 0.22 | -0.88 (-3.13, 1.36) | 0.43 | |  |
| BMI | 1.42 (0.39, 2.44) | **0.008** | 1.71 (0.71, 2.72) | **0.001** | |  |
| Male | -3.73 (-9.67, 2.20) | 0.28 | -3.50 (-8.96, 1.96) | 0.20 | |  |
| C-Reactive Protein | 0.04 (-0.01, 0.08) | 0.09 | 0.002 (-0.04, 0.05) | 0.09 | |  |
| Smoking | -3.33 (-9.05, 2.38) | 0.25 | -2.01 (-7.05, 3.03) | 0.43 | |  |
| Creatinine | -0.44 (-2.74, 1.86) | 0.70 | -0.51 (-2.68, 1.65) | 0.63 | |  |
| **Pyrazinamide AUC_0-24_** |  |  |  |  | |  |
| HIV positive | -5.18 (-47.64, 37.27) | 0.81 | 2.45 (-53.44, 48.54) | 0.92 | |  |
| Age (per 10 years) | -5.82 (-22.83, 11.19) | 0.50 | -0.25 (-19.63, 19.13) | 0.98 | |  |
| BMI | -0.54 (-8.52, 7.45) | 0.89 | -2.74 (-11.38, 5.89) | 0.53 | |  |
| Male | -33.67 (-76.99, 9.66) | 0.12 | -41.09 (-88.11, 5.92) | 0.08 | |  |
| C-Reactive Protein | -0.06 (-0.39, 0.28) | 0.73 | -0.06 (-0.45, 0.32) | 0.75 | |  |
| Smoking | -33.49 (-75.06, 8.09) | 0.11 | -31.21 (-74.63, 12.20) | 0.15 | |  |
| Creatinine | -8.84 (-25.59, 7.91) | 0.29 | -8.20(-26.83, 10.43) | 0.38 | |  |
| **Ethambutol AUC_0-24_** |  |  |  |  | |  |
| HIV positive | 1.27 (-1.27, 3.82) | 0.32 | 3.06 (-0.05, 6.07) | **0.046** | |  |
| Age (per 10 years) | 1.09 (0.97, 2.08) | **0.03** | 1.44 (0.29, 2.58) | **0.01** | |  |
| BMI | 0.01 (-0.47, 0.50) | 0.96 | -0.12 (-0.63, 0.39) | 0.63 | |  |
| Male | 0.55 (-2.12, 3.21) | 0.68 | -0.83 (-3.61, 1.94) | 0.55 | |  |
| C-Reactive Protein | -0.01 (-0.03, 0.01) | 0.41 | -0.01 (-0.04, 0.01) | 0.21 | |  |
| Smoking | -0.86 (-3.42, 1.70) | 0.50 | -0.30 (-2.90, 2.31) | 0.82 | |  |
| Creatinine | -0.01 (-1.18, 0.86) | 0.41 | -0.61 (-1.72, 0.50) | 0.28 | |  |

*Note: Univariate and multivariable β coefficients represent the change in AUC0-24 for a 1-unit increase in each predictor, adjusted for the other variables in the multivariable analysis.

| **Table S3** Determinants of AUC_0-24_ of antiTB drugs at week-6 by univariable and multivariable analyses | | | | |  | |
| --- | --- | --- | --- | --- | --- | --- |
| **Variable** | **Univariable β* (95% CI)** | **p, value** | **Multivariable β* (95% CI)** | **p-value** | |  |
| **Rifampicin AUC_0-24_ at week-6** |  |  |  |  | |  |
| HIV positive | 0.02 (-7.27, 7.31) | 0.99 | 1.53 (-6.21, 9.28) | 0.69 | |  |
| Age (per 10 years) | -0.05 (-2.99, 2.89) | 0.97 | 1.59 (-1.34, 4.53) | 0.28 | |  |
| BMI | 1.33 (-0.01, 2.66) | **0.05** | 0.93 (-0.39, 2.25) | 0.16 | |  |
| Male | -12.68 (-19.26, -6.11) | **<0.001** | -13.75 (-20.82, -6.67) | **<0.001** | |  |
| C-Reactive Protein | 0.02 (-0.03, 0.08) | 0.42 | 0.02 (-0.03, 0.08) | 0.38 | |  |
| Smoking | -0.80 (-8.04, 6.43) | 0.82 | -1.15 (-7.76, 5.45) | 0.73 | |  |
| Creatinine | -0.64 (-3.43, 2.14) | 0.64 | -0.89 (-3.67, 1.89) | 0.52 | |  |
| **Isoniazid AUC_0-24_ at week-6** |  |  |  |  | |  |
| HIV positive | 2.22 (-1.57, 6.02) | 0.24 | 2.42 (-1.81, 6.65) | 0.26 | |  |
| Age (per 10 years) | -1.29 (-2.80, 0.22) | 0.10 | -0.91 (-2.51, 0.69) | 0.26 | |  |
| BMI | 0.64 (-0.07, 1.35) | 0.08 | 0.65 (-0.06, 1.37) | 0.07 | |  |
| Male | -3.61 (-7.40, 0.17) | 0.06 | -3.14 (-7.00, 0.72) | 0.11 | |  |
| C-Reactive Protein | 0.01 (-0.02, 0.04) | 0.53 | 0.00 (-0.03, 0.03) | 0.98 | |  |
| Smoking | -3.79 (-7.46, -0.12) | **0.04** | -3.44 (-7.05, 0.17) | 0.06 | |  |
| Creatinine | -0.61 (-2.07, 0.85) | 0.40 | -0.31 (-1.83, 1.20) | 0.68 | |  |
| **Pyrazinamide AUC_0-24_ at week-6** |  |  |  |  | |  |
| HIV positive | 36.40 (-25.17, 97.96) | 0.24 | 31.26 (-35.09, 98.62) | 0.35 | |  |
| Age (per 10 years) | -11.20 (-36.18, 13.78) | 0.37 | -4.58 (-30.09, 20.93) | 0.71 | |  |
| BMI | 16.21 (5.21, 27.21) | **0.005** | 15.38 (3.91, 26.85) | **0.01** | |  |
| Male | -49.18 (-111.31, 12.95) | 0.12 | -44.60 (-106.10, 16.89) | 0.15 | |  |
| C-Reactive Protein | 0.50 (0.02, 0.98) | **0.04** | 0.39 (-0.12, 0.90) | 0.13 | |  |
| Smoking | -24.89 (-86.43, 36.64) | 0.42 | -20.80 (-78.30, 36.69) | 0.47 | |  |
| Creatinine | -10.20 (-33.90, 13.50) | 0.39 | -7.71 (-31.89, 16.47) | 0.52 | |  |
| **Ethambutol AUC_0-24_ at week-6** |  |  |  |  | |  |
| HIV positive | -1.64 (-5.12, 1.85) | 0.35 | -0.08 (-4.28, 4.13) | 0.97 | |  |
| Age (per 10 years) | 0.25 (-1.16, 1.67) | 0.72 | 0.62 (-0.97, 2.21) | 0.44 | |  |
| BMI | 0.43 (-0.23, 1.09) | 0.20 | 0.20 (-0.51, 0.92) | 0.57 | |  |
| Male | -2.32 (-5.85, 1.19) | 0.19 | -2.82 (-6.67, 1.01) | 0.15 | |  |
| C-Reactive Protein | -0.01 (-0.03, 0.02) | 0.68 | -0.001 (-0.03, 0.03) | 0.97 | |  |
| Smoking | 0.03 (-3.46, 3.52) | 0.98 | 0.37 (-3.22, 3.96) | 0.83 | |  |
| Creatinine | -1.15 (-2.45, 0.16) | 0.08 | -1.18 (-2.69, 0.33) | 0.12 | |  |

*Note: Univariate and multivariable β coefficients represent the change in AUC0-24 for a 1-unit increase in each predictor, adjusted for the other variables in the multivariable analysis.
